# Novel Polyelectrolyte-Based Draw Solute That Overcomes the Trade-Off between Forward Osmosis Performance and Ease of Regeneration

**DOI:** 10.3390/membranes12121270

**Published:** 2022-12-15

**Authors:** Daryoush Emadzadeh, Amirsajad Atashgar, Boguslaw Kruczek

**Affiliations:** Department of Chemical and Biological Engineering, University of Ottawa, Ottawa, ON K1N 6N5, Canada

**Keywords:** draw solute, forward osmosis, regeneration, ultrafiltration

## Abstract

Forward osmosis (FO) is an emerging technology for seawater and brackish desalination, wastewater treatment, and other applications, such as food processing, power generation, and protein and pharmaceutical enrichment. However, choosing a draw solute (DS) that provides an appropriate driving force and, at the same time, is easy to recover, is challenging. In this study, water-soluble poly(styrene sulfonate) (PSS) was modified by a high-electrical-conductivity 3,4-ethylenedioxythiophene (EDOT) monomer to fabricate a novel draw solute (mPSS). FO tests with the CTA membrane in the active layer facing the feed solution (AL-FS) orientation, using a 50 mS/cm aqueous solution of synthesized solute and distilled water as a feed solution exhibited a water flux of 4.2 L h^−1^ m^−2^ and a corresponding reverse solute flux of 0.19 g h^−1^ m^−2^. The FO tests with the same membrane, using a 50 mS/cm NaCl control draw solution, yielded a lower water flux of 3.6 L h^−1^ m^−2^ and a reverse solute flux of 4.13 g h^−1^ m^−2^, which was more than one order of magnitude greater. More importantly, the synthesized draw solute was easily regenerated using a commercial ultrafiltration membrane (PS35), which showed over 96% rejection.

## 1. Introduction

Forward osmosis (FO) is a novel technology that utilizes the natural phenomenon of osmosis. It has shown promising potential in various applications, including seawater/ brackish desalination [1,2], wastewater treatment [3,4], food processing [5,6], power generation [7], and protein and pharmaceutical enrichment [8,9]. Compared to pressure-driven membrane processes like reverse osmosis (RO) and nanofiltration (NF), FO offers savings in energy consumption, reductions in membrane fouling [10,11], and a high rejection of many contaminants [10]. The FO membrane should minimize internal concentration polarization (ICP) and exhibit high solute rejections. The development of FO membranes [1,12,13] and the design of the process [14] have been thoroughly investigated in the literature. Another critical aspect, which has received less attention than membrane development [15], is the selection of a draw solute (DS) [16]. 

Preferable draw solutes are expected to have the following characteristics: (1) high osmotic pressure, which can result in a high water flux; (2) minimal reverse-draw-solute flux; and (3) a diluted DS, which should be easily recoverable [17]. Additionally, draw solutes must be nontoxic, relatively inexpensive, and compatible with the FO membrane. In recent years, inorganic salt draw solutes have gained a lot of attention, such as ammonium carbonate [18], fertilizers [19], and magnetic nanoparticles [20]. Among these inorganic draw solutes, ammonium carbonate shows excellent potential because it has high osmotic pressure and is relatively easy to regenerate. However, its recycling technique is expensive in energy consumption. 

Synthetic draw solutes, such as magnetic nanoparticles, ferric and cobaltous hydro acid complexes, switchable polarity solvents, and 2-methylimidazole-based compounds, have been found to have minimal reverse solute fluxes and low-energy consumption during regeneration [21]. Moreover, these nanoparticles can generate high osmotic pressure [22]. The efficient regeneration of magnetic nanoparticles via heat-facilitated magnetic separation is a distinct advantage. However, the particles will likely agglomerate via magnetic or electric separators during the recycling process. Another significant drawback of magnetic nanoparticles is their complicated synthesis and the lack of experience testing them on a large scale. Consequently, further research is needed to develop and investigate this family of draw solutes.

Fertilizers could be used as a draw solute, and diluted fertilizers may be used in fertilization after the FO process without needing recovery, which is a great advantage. This is, however, limited to agriculture applications [23]. Recently, some studies have also widely examined stimuli-responsive polymer-hydrogel draw solutes because of their low-energy regenerative process [24,25]. At the same time, water flux, when using them as a draw solute, is low. Another approach is to use switchable polarity solvents as a draw solution. Wilson and coworkers [26] demonstrated that switchable polarity solvents could be mechanically separated from purified water after the polar to nonpolar phase shift. However, cellulose triacetate (CTA) FO membranes can be degraded in their application. Different possible draw solute candidates, including organic ionic salt [21] and organic compounds [27], have also been investigated. In general, draw solutes encounter the trade-off between high osmotic pressure and easy regeneration, and there is no draw solute that fulfills all the criteria mentioned earlier [28]. 

Recently, poly(sodium4-styrene sulfonate) (PSS) polyelectrolytes have been employed for the FO system. Because of its high molecular weight, PSS can be recovered in an ultrafiltration (UF) process. Although the osmotic pressure generated by PSS is relatively high, it is not high enough to generate attractive water fluxes [17]. Therefore, PSS is another trade-off between high osmotic pressure and easy regeneration.

Poly(3,4-ethylene dioxythiophene) (PEDOT) is one of the most studied conductive polymers (CP) due to its relatively high-electrical-conductivity and electro-optical properties [28]. It can be easily synthesized via oxidative chemical and electrochemical polymerization of its monomer without requiring any specific setup [29]. For example, hybrid hydro-responsive actuators were developed by infiltrating carbon nanotube yarns using poly (3,4-ethylenedioxythiophene):poly(styrenesulfonate). These actuators are very responsive to water humidity changes and show extra capability of electrical actuation. As a result, they show great potential for artificial muscles, hydro-driven generators, moisture switches, and microfluidic mixers [30]. Although PEDOT has high electrical conductivity, it is insoluble in water, making it impossible to use it as a draw solute. However, its monomer, 3,4-ethylenedioxythiophene (EDOT), is water-soluble and can be combined with PSS via an oxidation technique [31]. In their study, Sakunpongpitiporn et al. considered the effects of EDOT:PSS and EDOT: Na_2_S_2_O_8_ ratios with and without the presence of different surfactants on the properties of the resulting PEDOT:PSS and, in particular, their electrical conductivity. However, they did not report the water solubility of the synthesized nanoparticles.

In this study, we adapted the protocol used for combining EDOT:PSS via an oxidation technique [31]. However, we modified it by using FeSO_4_**·**7H_2_O as a catalyst to achieve the water-soluble product. To distinguish our product from nanoparticles synthesized by Sakunpongpitiporn et al. [31], we denoted it as modified PSS (mPSS). We compared the water and reverse solute fluxes in FO experiments using draw solutions based on mPSS and NaCl, respectively, of the same electrical conductivity. We then demonstrated the possibility of the regeneration of an mPSS-based draw solution using a commercial PS35 UF membrane. 

## 2. Materials and Methods

### 2.1. Materials

Liquid poly(sodium4-styrene sulfonate) (PSS) with a molecular weight of 75,000 g.mol^−1^ and 99% purity was purchased from Sigma-Aldrich, (Saint Louis, MO, USA). The 3,4-Ethylenedioxythiophene (EDOT, 97% purity) monomer, sodium persulfate, Na_2_S_2_O_8_, (98% purity), and FeSO_4_·7H_2_O were procured from Sigma Aldrich. Reagent-grade acetone, methanol, and sodium chloride were purchased from Fisher Scientific, (Hampton, NH, USA). Deionized water was used in chemical reactions, and distilled water was used to prepare the synthetic brackish water and draw solutions. Poly(sulfone) ultrafiltration membranes (PS35) with a molecular weight cut-off of 20 kDa and an average pore size of 17 nm were donated by Solecta, (Oceanside, CA, USA). Flat-sheet cellulose triacetate (CTA) membranes were procured from Sterlitech Corporation, (Auburn, WA, USA).

### 2.2. Synthesis of mPSS

Modified PSS was synthesized in the reaction of PSS with EDOT. A total of 0.5 g of EDOT was mixed with 6.5 g PSS and 0.8335g of oxidant Na_2_S_2_O_8_. Therefore, the molar ratio of EDOT:Na_2_S_2_O_8_ was 1:1. In addition, 0.2 wt% of FeSO_4_·7H_2_O was used as a catalytic agent. The ingredients were dissolved in 100 mL of DI water, and then the solution was stirred for 24 h at room temperature. After that, the solution content was centrifuged at 6000 rpm for 15 min. The collected precipitate, mPSS, was rinsed with an acetone/methanol solution with a volume ratio of 3:20 [31]. The collected precipitate was air-dried, followed by overnight drying in an oven at 90 °C. The final product was a dark-blue powder.

### 2.3. Characterization of the Synthetic Draw Solute 

The ATR-FTIR spectra of the mPSS draw solute were obtained using a Nicolet 6700 FTIR (Thermo Scientific) with a diamond crystal. The draw solute was also investigated using X-ray diffraction (Rigaku Ultima IV Diffractometer, from Rigaku Corporation, Tokyo, Japan). The XRD tests were performed at room temperature using Cu K radiation (wavelength *λ* =1.5418) with Bragg–Brentano geometry. The 2*θ* range of 4° to 70° was covered with a 0.02° step width and a 3°/min scan speed. The XRD spectra were used, along with the Scherrer equation, to determine the average crystallite size of the mPSS particles [32].

(1) $$D=\frac{0.9\lambda }{\beta \cos\theta }$$

where *D* is the average crystallite size, *θ* is Bragg’s angle, and *β* is the full width at half maximum (radian). 

A tabletop scanning electron microscope (Tescan Vega-II XMU from Tescan, Brno – Kohoutovice, Czech Republic), equipped with an energy-dispersive X-ray spectroscope, was used to study the mPSS draw solute morphology and identify atoms on the surface.

### 2.4. Evaluation of Membrane Performance

The performance of the mPSS draw solute was evaluated in FO tests using a CTA membrane in the AL-FS orientation. The details of the testing system are described elsewhere [33]. The synthesized mPSS was dissolved in DI water to form the draw solution of a concentration of 175 g/L, with an electrical conductivity of 50 mS/cm. For comparison, a draw solution of the same electrical conductivity (50 mS/cm) was also prepared using NaCl (28.2 g/L). As a feed solution, we used distilled or synthetic brackish water (3.67 g/L) with an electrical conductivity of 7 mS/cm. The FO system yielded the continuous monitoring of the mass of the feed and draw solutions using high-resolution balances (0.01 g for the feed solution and 0.1 g for the draw solution) connected to a personal computer equipped with Lab View data-acquisition software. In addition, the conductivity and temperature of the feed solution were also recorded continuously using a benchtop conductivity/temperature meter, T-C (CON2700, Oakton Instruments, Woonsocket, RI, USA). To minimize the effects of external concentration polarization, the feed and draw solutions were circulated at 2.4 L/min, i.e., at the maximum circulation rate allowed by the respective centrifugal pumps (TE-3-MD-HC, Little Giant Co., Calhoun, GA, USA). All experiments were performed at 24 °C. Each FO test was carried out for at least 30 min, which was more than enough time to reach steady-state conditions. The latter was signified by the constant rate of water mass change (*dm_w_/dt*) at the draw and feed sides of the membrane. Consequently, the water flux was evaluated from:

(2) $$J_{w}=\frac{dm_{w}/dt}{\rho A_{m}}$$

where *ρ* is the density of water and *A_m_* is the membrane area (20.6 cm^2^). At a steady state, the rate of conductivity change in the feed solution was constant. Using in-house-prepared calibration curves for the mPSS and NaCl solutions, the measured conductivity was converted into the concentrations of the respective solutes in the feed solution. In turn, knowing the mass of the feed, the product of the feed solution’s concentration and the feed solution’s corresponding mass yielded an assessment of the rate of change of the mass of the draw solute in the feed solution (*dm_s_/dt*), which was used to evaluate the reverse-draw-solute flux:

(3) $$J_{s}=\frac{dm_{s}/dt}{A_{m}}$$

The reverse-draw-solute flux was evaluated only in the experiments using distilled water as a feed solution. 

The volumes of the draw (700 mL) and feed solution (400 mL) in each experiment were much larger than the total volume of water transferred during the entire experiment. Similarly, the concentration of the draw solute in the feed solution at the end of each experimental run was much smaller than the initial concentration of the draw solution. Consequently, the respective driving forces for transporting water and the draw solute remained practically constant during the entire FO test. 

Batch UF experiments were conducted in a stainless-steel self-stirred membrane permeation cell using the commercial PS35 ultrafiltration membrane. The tests were performed with different conductivities (concentrations) of mPSS feed solution (5–50 mS) at pressures from 10 to 140 psig and at an ambient temperature. For comparison, we tested NaCl solutions with similar conductivities to the mPSS solutions. All tests were carried out using 200 mL of feed solution stirred at 350 rpm to reduce the effects of concentration polarization. The tests were also performed with pure water.

Water flux (*J_w_*) and solute rejection (*R*) were calculated from:

(4) $$J_{w}=\frac{\Delta V}{A_{m}\Delta t}$$

where *A_m_* is the effective membrane area (11.4 cm^2^), Δ*V* is the permeate volume, and Δ*t* is the time. In each test, the collected Δ*V* was the same and was equal to 50 mL, i.e., 25% of the original volume of the feed.

(5) $$R=\left(1-\frac{C_{p}}{C_{f}}\right)\times 100$$

where *C_f_* is the initial feed concentration and *C_p_* is the solute concentrations in the first 50 mL of the collected permeate. Considering that the concentration of solute in the feed increases as water permeates through the membrane, the solute rejection calculated from Equation (5) may slightly underestimate the true *R* of the membrane.

## 3. Results and Discussion

### 3.1. Characterization of the mPSS Draw Solute 

**Scheme 1:**
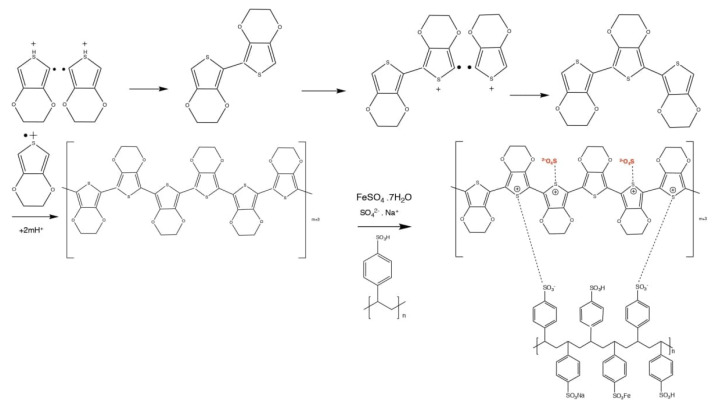
Chemical oxidative polymerization of PSS and EDOT in the presence of sodium persulfate oxidant and FeSO_4_·7H_2_O catalyst.

The chemical oxidative polymerization reaction between EDOT and PSS is depicted in Scheme 1. The oxidant, Na_2_S_2_O_8_, first dissociates into persulfate and sodium ions. The persulfate ions in an aqueous solution can then break down homiletically into sulphate radicals. The EDOT monomer was oxidized into an EDOT radical cation by the sulphate radical during the oxidation stage, resulting in the creation of sulphate anions. The EDOT radical cations create two protons in the propagation and doping step, which can be eliminated in this step. Sulphate ions and PSS, which act as dopants during polymerization, can interact with the oxidized PEDOT chains at the same time. As seen in Scheme 1, the sodium ion from the oxidant can react with the PSS chains.

**Figure 1:**
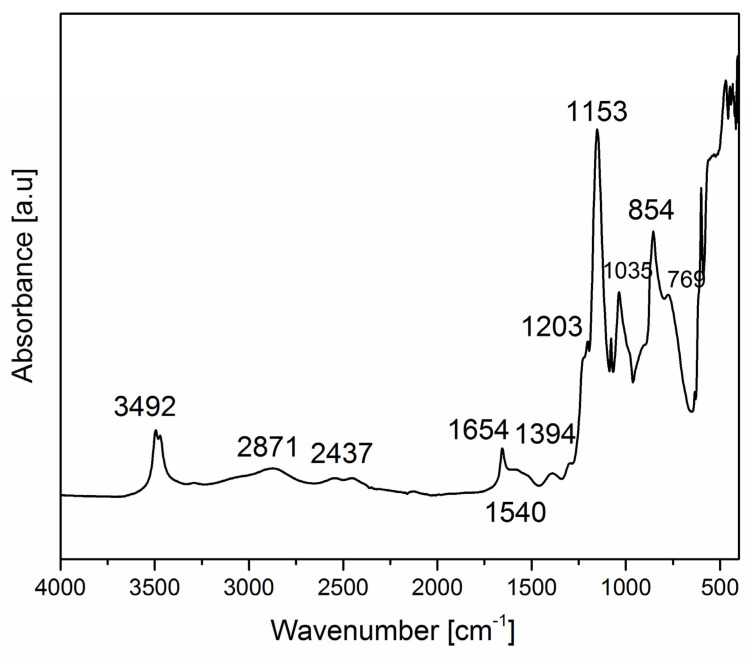
FTIR spectrum of lab-synthesized mPSS.

Figure 1 shows the FTIR of mPSS with some particular functional groups. There are two types of C=C stretching in the aromatic rings of PSS and the thiophene ring of PEDOT, which are located at 1654 and 1540 cm^−1^. In addition, the peak at 1394 cm^−1^ is related to the C–C stretching in the PEDOT thiophene ring [32]. The peak at 769 cm^−1^ is assigned to the C–S stretching of the thiophene ring in PEDOT. The peak at 1203 and 853 cm^−1^ are assigned to the symmetric stretching and S–phenyl bond in PSS [34]. The peaks at 1153 cm^−1^ can be attributed to SO_4_^2-^ from the oxidant [35]. The resulting peaks from PSS incorporated in the PEDOT chain appear at 3492 and 2871 cm^−1^ and are assigned to the O–H stretching of PSS and the C–H stretching of PEDOT, respectively. These peaks confirm the formation of a draw solute with appropriate functional groups based on their reaction [35]. 

**Figure 2:**
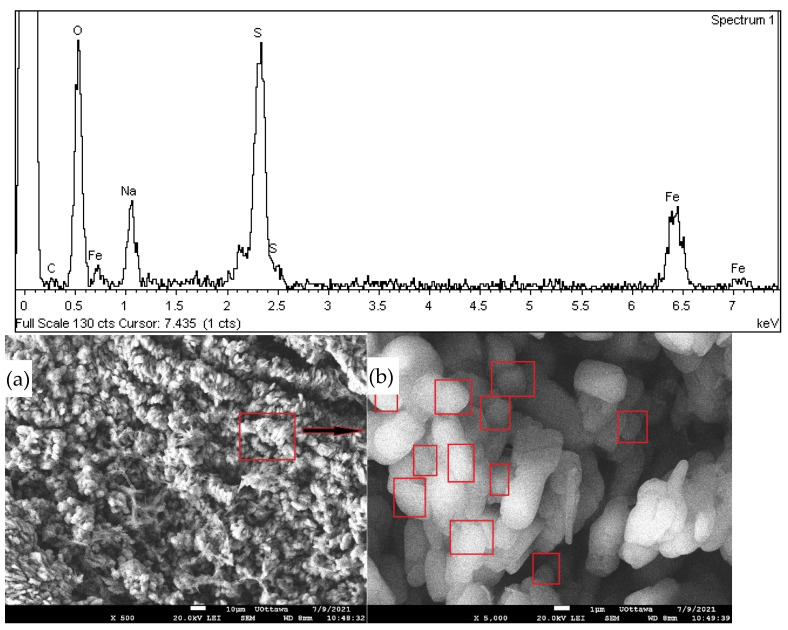
EDX analysis and SEM images and of the mPSS solute particles. The right image (**b**) is a magnification of the red rectangle in the left image (**a**).

Figure 2 displays the SEM image and the summary of the EDX of the synthetic mPSS draw solute. The EDX analysis reveals carbon, oxygen, and sulfur as the main elements of the 3,4 ethylenedioxythiophene monomer and poly(styrene sulfonate). In addition, Na and Fe elements can be attributed to the Na_2_S_2_O_8_ oxidant and FeSO_4_·7H_2_O catalyst, respectively. In general, the particles appear to be rectangular in the SEM image.

**Figure 3:**
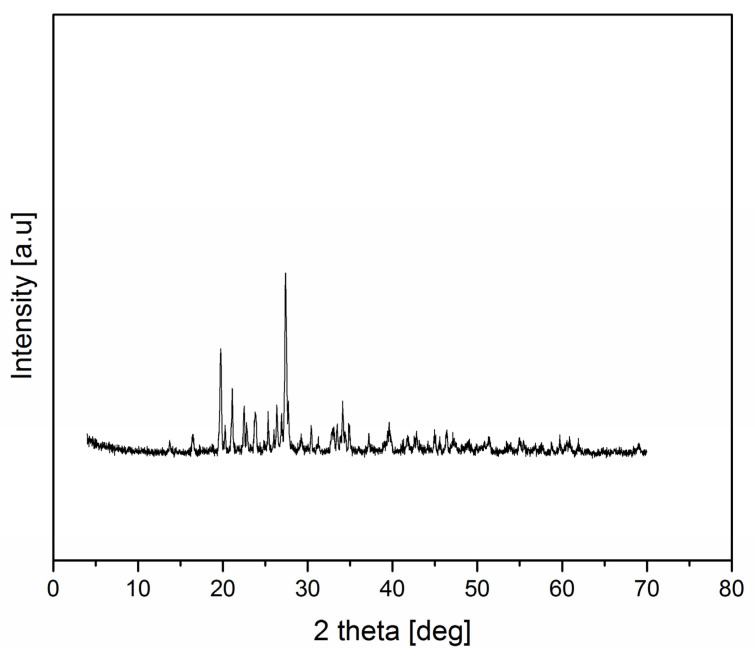
The XRD spectrum of lab-synthesized mPSS.

The XRD spectrum of mPSS is shown in Figure 3. The PSS and inter-chain packing of PEDOT is shown by the two prominent peaks at 19.7 and 27.3, respectively [34]. Furthermore, the broad diffraction peak at 2*θ* = 27.3° suggests that the crystallization of the PEDOT-PSS conductive solute was in the amorphous phase, which is consistent with the literature [34]. According to Equation (1), the solute’s average crystallite size is 39.8 nm.

### 3.2. Evaluation of the Draw Solute in FO Tests

The FO tests were performed using a commercial CTA membrane in the AL-FS orientation. The lab-synthesized draw solution was compared to the aqueous NaCl solution. The draw solutions had the same conductivity of 50 mS/cm. The tests were performed with distilled and synthetic brackish water (conductivity of 7 mS/cm). Three independent tests were performed for each combination of the draw and feed solutions using a new CTA membrane in every test. Table 1 compares the water flux of both draw solutions obtained with pure and brackish water as a feed. The corresponding reverse salt fluxes of both draw solutes with pure water as a feed is also shown in Table 1.

It can be noticed that there is some variation between the tests in identical conditions. This is likely due to using a new CTA membrane coupon in each test. Although the CTA membrane is a commercial product, it does not guarantee identical properties of relatively small membrane coupons cut from different locations of the membrane sheet [36]. Therefore, in the following discussion, we will focus on the average values from three independent tests. 

For both feed solutions, the water flux when using mPSS as a draw solution was slightly greater than that of a NaCl draw solution of the same conductivity. In both cases, the water flux with pure water as a feed was also slightly larger than that the water flux with the synthetic brackish water as a feed. This is not surprising because the presence of NaCl in the feed decreases the osmotic pressure gradient. Assuming that osmotic pressure is directly proportional to the electrical conductivity of the solution, a decrease in water flux when replacing the water with synthetic brackish water should be approximately 14%. However, the observed decrease in the water flux was less than 14%. More specifically, it was 3.5% for the lab-synthesized draw solution and 10% for the NaCl draw solution. Therefore, although the osmotic pressure increased with the electrical conductivity, there was no direct proportionality between the two parameters [17]. The lack of direct proportionality between the electrical conductivity and the osmotic pressure was further indicated by the higher water flux resulting from the lab-synthesized draw solution —4.2 L/m^2^ h versus 3.6 L/m^2^—for the NaCl solution of the same conductivity (50 mS/cm). It is, therefore, evident that the mPSS solution creates more significant osmotic pressure than the NaCl solution. Although NaCl is a strong electrolyte, the number of ions contained in the mPSS draw solution is substantially greater than that in the NaCl solution with the same molar concentration [17], which is responsible for the more significant osmotic pressure of the former.

The water flux results in Table 1 illustrate the superiority of the mPSS draw solution. However, the main advantage of the lab-synthesized mPSS is a reverse solute flux that is an order of magnitude smaller than NaCl (0.19 g/m^2^ h versus 4.13 g/m^2^ h). The FO tests were performed at nearly zero stage-cut conditions, i.e., at a practically constant driving force. If the experiments were carried out for a prolonged time, or using a much larger membrane area, a reverse salt flux of NaCl that is greater than one order of magnitude would lead to a significant decrease in osmotic pressure and, ultimately, water flux. As already indicated, mPSS particles have an average crystallite size of 39.8 nm, which is much greater than 0.3 nm, i.e., the pore size of the CTA membrane [37]. This is the main reason for the minimal reverse flux of the mPSS draw solute. On the other hand, despite such a small pore size, the CTA membrane does not effectively reject very small NaCl solutes.

### 3.3. Regeneration of Draw Solution Test by UF Membrane

In practical FO applications, the draw solution concentration becomes increasingly diluted because of water transport from the feed side and the reverse solute flux, also referred to as draw solute leakage to the feed side. Although the reverse solute flux of the synthesized mPSS is minimal, it will still be diluted because of the water flux from the feed solution. Therefore, for a continuous operation, the FO process must be combined with the simultaneous regeneration of the draw solution [38]. In other words, FO is not typically a stand-alone process but rather a hybrid process. The attractiveness of a hybrid FO process depends on the regeneration of the draw solution. The application of size-exclusive regeneration processes, particularly UF membranes, helps the hybrid FO process be energy efficient [39,40]. Because of the relatively high molecular weight of base PSS, we considered using commercial PS35 ultrafiltration to concentrate the dilute solution of mPSS. 

**Figure 4:**
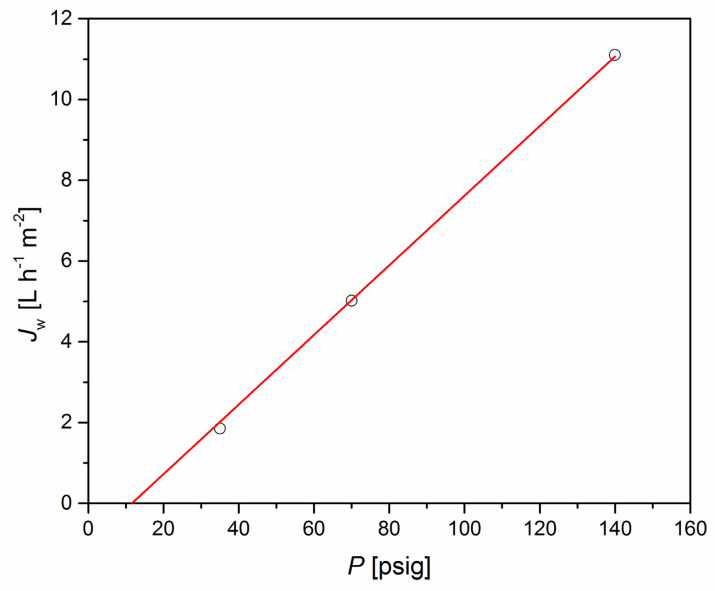
Water flux as a function of pressure in batch separation experiments with a 5 mS/cm mPSS solution using PS35 UF membrane.

Figure 4 presents the water as a function of applied pressure in batch experiments using a dilute solution of mPSS (conductivity of 5 mS) with a PS35 UF membrane. Similarly to the FO experiments, we used a new membrane coupon in every test. 

As expected, *J_w_* is a linear function of Δ*P*. However, the intercept of *J_w_* vs Δ*P* is not zero. Because PS35 can reject mPSS, the draw solution generates osmotic pressure even at a relatively low concentration. The intercept with the pressure axis, i.e., *J_w_* = 0, can be considered the magnitude of osmotic pressure. Accordingly, for a 5 mS mPSS solution in contact with the PS35 UF membrane, osmotic pressure is approximately 12 psig. At the highest applied pressure of 140 psig, the corresponding water flux is 11.1 L h^−1^ m^−2^.

**Figure 5:**
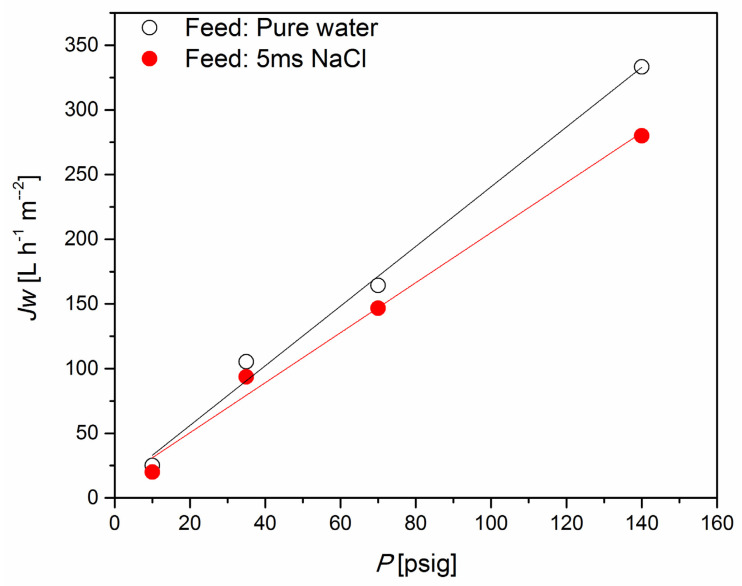
Water flux as a function of pressure in batch permeation/separation experiments with a 5 mS/cm NaCl aqueous solution and pure water using PS35 UF membrane.

We also performed comparative UF experiments with a 5 mS/cm NaCl solution and distilled water using a PS35 UF membrane. The results of these tests are displayed in Figure 5.

Similar to Figure 4, water flux increased linearly with applied pressure. However, unlike Figure 4, the intercept of *J_w_* vs Δ*P* was minimal for both pure water and the dilute solution of NaCl with water. For a given Δ*P*, the pure water flux was greater than that of the NaCl aqueous solution, and both were much greater than the water flux using a 5 mS/cm solution of mPSS as a feed (Figure 4). 

The lack of intercept in the tests with the NaCl solution indicates that it cannot generate osmotic pressure in contact with the PS35 UF membrane. It also implies that the PS35 UF membrane does not reject NaCl, which is not surprising considering the membrane’s pore size, which is much greater than the size of the solute. Without applied pressure, a NaCl concentration gradient between the feed and permeate side of the PS35 UF membrane would not be maintained because of the diffusion of the solute through water-filled pores of the membrane.

Figure 4 shows that water flux associated with the separation of the 5 mS/cm mPSS solution using the PS35 UF membrane was reasonably high, particularly at 140 psig. However, in the actual regeneration of a draw solution, it is necessary to concentrate the draw solution to the level used in the FO process. In our case, the data reported in Table 1 were obtained using a 50 mS/cm mPSS solution. Can the osmotic pressure generated by a 50 mS/cm mPSS solution in contact with PS35 UF be overcome using a moderate pressure gradient? In Figure 4, the highest applied pressure was 140 psig, and we will use this pressure to answer our question. Notably, 140 psig, nearly 10 bar, is closer to typical pressures in nanofiltration than in ultrafiltration processes. However, as we already demonstrated, the commercial PS35 UF membranes can easily withstand such applied pressure.

**Figure 6:**
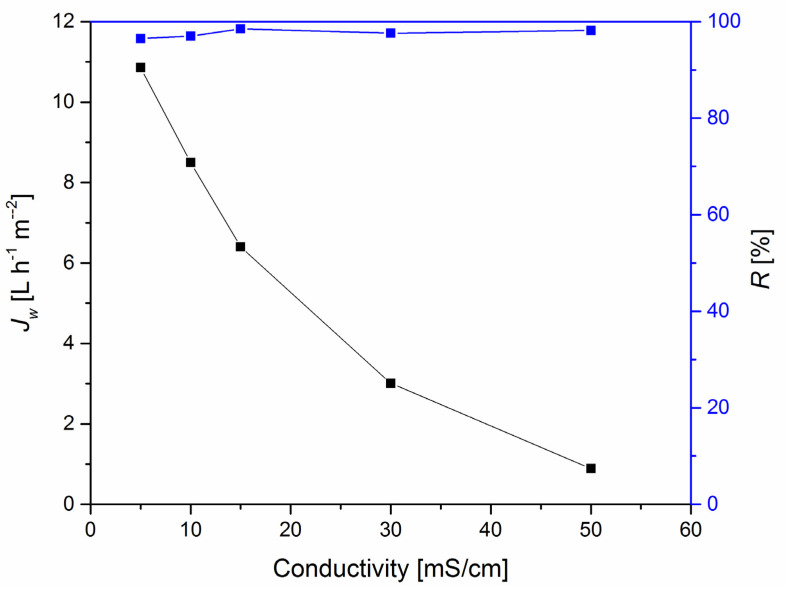
Water flux and rejection of mPSS solute as a function of conductivity (concentration) of mPSS solution using commercial PS35 UF membrane at 140 psig.

Figure 6 presents water flux and solute rejection as a function of the concentration (conductivity) of the mPSS solution at 140 psig. Not surprisingly, as the conductivity of the mPSS solution increased, the water flux decreased. However, the relationship between the water flux and the conductivity is not direct inverse proportionality. If it were, there would be no water flux at the highest conductivity of the mPSS solution. Although small (0.89 L h^−1^ m^−2^), there was still water flux with the 50 mS/cm mPSS feed solution. In other words, the osmotic pressure generated by the 50 mS/cm mPSS solution in contact with the PS35 UF membrane must be less than 140 psig. In addition, as explained in Section 2.3., *J_w_* and *R* are based on the first 50 mL of the permeate, representing 25% of the initial feed solution. Consequently, because PS35 UF rejects the mPSS solute, the feed solution’s effective conductivity (concentration) for 0.89 L h^−1^ m^−2^ must be greater than 50 mS/cm. 

Regarding the rejections of mPSS by the PS35 UF membrane, they were greater than 96% for any conductivity of the feed solution. The rejection slightly increased as the conductivity increased from 5 to 15 mS/cm at plateaus at nearly 98% at larger conductivities of the feed solution. As explained in Section 2.3., because of the batch nature of the UF experiments, the actual rejections of mPSS by PS35 UF might be slightly greater than those shown in Figure 6. 

The results in Figure 6 indicate that the mPSS draw solution can be regenerated to 50 mS/cm initial conductivity, and high-quality water can be produced simultaneously using the commercial PS35 UF membranes. Therefore, the lab-synthesized draw solute based on high molecular PSS can overcome the trade-off between the FO water flux and the ease of regeneration of the draw solution, signified by applying a UF membrane. At the same time, another trade-off became evident in this study. The high quality of water produced while regenerating the mPSS draw solution implies that, although a UF membrane can be used, because of the high rejection of the draw solute, the osmotic pressure that must be overcome is also relatively large. Consequently, typical UF pressures are insufficient for solute regeneration, and “nanofiltration pressures” are required. Still, the fact that the regeneration of the draw solution is possible using a UF membrane instead of NF or RO membranes offers a great advantage to the mPSS synthesized in this work. It is also important to note that the FTIR analysis of mPSS recovered from the concentrate in the regeneration process using the PS35 membrane did not indicate any significant difference in structure. This suggests the stability of the draw solute at the operating conditions used in our work, particularly during its regeneration at 10 bar. 

Table 2 compiles information about different draw solutes from the literature and lab-synthesized mPSS in this work, focusing on their chemical nature, regeneration method(s), advantages, disadvantages, and the range of the water and reverse solute fluxes. The direct comparison of the water and reverse solute fluxes of the entries in Table 2 is problematic because they were tested at different concentrations of the respective draw solutions. In the case of some entries in Table 2, the conditions used in the FO tests are not available. Keeping in mind this limitation, the FO performance of mPSS is on par with EDTA sodium salt draw solute. However, the latter is regenerated using the nanofiltration (NF) or direct-contact membrane-distillation (DCMD) process. Although mPSS requires nanofiltration pressures in its regeneration, UF membranes, such as PS35, are sufficient in our case, whereas the recovery of EDTA sodium salt draw solute requires an NF membrane. 

## 4. Conclusions

We successfully synthesized a novel draw solute based on a poly (sodium 4-styrene sulfonate) (PSS) polyelectrolyte. PSS was reacted with EDOT. A total of 0.5 g of EDOT was utilized in the presence of an oxidant (Na_2_S_2_O_8_) and a catalyst (FeSO_4_·7H_2_O). The FT-IR analysis confirmed the chemical reaction. Modified PSS (mPSS) was characterized by XRD and SEM with EDX. The performance of mPSS was evaluated in FO experiments with pure and brackish water as a feed solution. The FO water flux generated by the mPSS draw solution was greater than that of the NaCl draw solution of the same electrical conductivity (4.2 L s^−1^ m^−2^ versus 3.6 L s^−1^ m^−2^). More remarkably, the corresponding reverse-draw-solute flux of mPSS was one order of magnitude smaller than that of NaCl (0.19 g s^−1^ m^−2^ versus 4.13 L s^−1^ m^−2^). We also demonstrated that the mPSS draw solution could be regenerated using commercial P35 UF membranes. The corresponding solute rejection was over 96%, indicating the possibility of producing pure water while regenerating the draw solution. The results of this study indicate that synthesized mPSS allows an overcoming of the trade-off between FO performance and the easy regeneration of the draw solution. 

## Figures and Tables

**Table 1:** Summary of the FO performance of CTA membrane operated in AL-FS orientation, using 50 ms/cm mPSS and NaCl as draw solutions and pure water and brackish water as feed solutions.

Draw/Feed	*J_w_* [L h^−1^ m^−2^]	*J_s_* [g h^−1^ m^−2^]
mPSS/pure water	4.6	0.24
	3.8	0.18
	4.2	0.15
	Avg. = 4.2	Avg. = 0.19
mPSS/brackish water	3.9	-
	4.4	-
	3.9	-
	Avg. = 4.1	-
NaCl/pure water	4.3	4.8
	3.1	4.3
	3.5	2.9
	Avg. = 3.6	Avg. = 4.13
NaCl/brackish water	2.9	-
	3.4	-
	3.3	-
	Avg. = 3.2	-

**Table 2:** Overview of draw solutes their recovery methods, advantages, disadvantages and FO performance.

Draw Solute	Regeneration Method(s)	Advantages	Disadvantages	*J_w_* [Lh^−1^ m^−2^]	*J_s_* [gh^−1^ m^−2^]	Ref.
NaCl, KCl	RO, thermal, membrane distillation (MD)	Low cost, high osmotic pressure, high solubility, less viscous	High reverse solute flux, high scaling/fouling tendency	8.1–11.8	4–15.3	[41,42]
(NH_4_)2HPO_4_, K_2_SO_4_, NaNO_3_, NH_4_H_2_PO_4_, K_2_SO_4_, NH_4_NO_3_, KNO_3_, KH_2_PO_4_	Not required	Diluted draw solution could be directly used in irrigation	High reverse solute flux, biofouling tendency is high	7.9–11.8	7–12	[43,44,45]
Iron (III) acetylacetonate + TEG + PAA, citrate-coated magnetic nanoparticles (cit-MNPs), dextran-coated Fe_3_O_4_ magnetic nanoparticles, PEG-(COOH)_2_-coated MNPs, PSA-MNPs, magneticthermo-responsive ionic nanogels	Magnetic separation	Easy recovery and reusability of NPs, high water flux obtained	Agglomeration of nanoparticles during recycling, magnetic properties of NPs may deteriorate	2.5–3.5	-	[46,47,48]
EDTA sodium salt, EDTA-2Na	NF, DCMD	Energy consumption is less, and high water flux is obtained, less reverse diffusion	Draw solute is much more expensive compared to standard salt	8.5	0.32	[49]
N-isopropyl acrylamide (NIPAM) and sodium acrylate hydrogels	Pressure and thermal stimuli	High water permeation rate, less back diffusion	Regeneration is not easy and highly viscous	0.2–1	-	[50]
Hydrolyzed polyacrylamide (HPAM)	-	Effective cleaning, least fouling	Expensive precursors	2.5–3.5	-	[51]
Sodium traethylenepentamine heptaacetate (STPH)	Recovery NF and Freezing Concentration (FC)	High water flux and very low reverse solute flux (PRO mode)	Highest salt leakage was obtained at pH = 10.	9.5	-	[52]
Poly(sodium styrene-4-sulfonate-co-N-opropylacrylamide) (PSSS-PNIPAM), poly (aspartic acid sodium salt) and 2- Methylimidazole compounds	Membrane distillation (MD)	Thermoresponsive property of the DS enhances the regeneration via MD, produces high-quality water	High operating cost in the regeneration process (membrane distillation)	7	2	[53]
Poly(sodium4-styrene sulfonate) (PSS) polyelectrolytes	UF	Higher osmotic pressure, higher viscosity	Reverse solute flux of this draw is still high, lower diffusion coefficient, more severe concentration polarization	10–11.5	6.8	[19]
mPSS = Poly(3,4-ethylene dioxythiophene):poly(styrene sulfonate)	UF	Produces high-quality water, negligible reverse draw solute, low cost, high osmotic pressure, high solubility, cost-effective regeneration	Viscous at high concentrations	4.2	0.19	This work

## Data Availability

The data presented in this study are available on request from the corresponding author.
